# Supplementary figures and images for: Paleoclimate data assimilation with CLIMBER-X: An ensemble Kalman filter for the last deglaciation
Source: PLoS One. 2024 Apr 4;19(4):e0300138. doi: 10.1371/journal.pone.0300138 (PMC10994341; doi:10.1371/journal.pone.0300138)

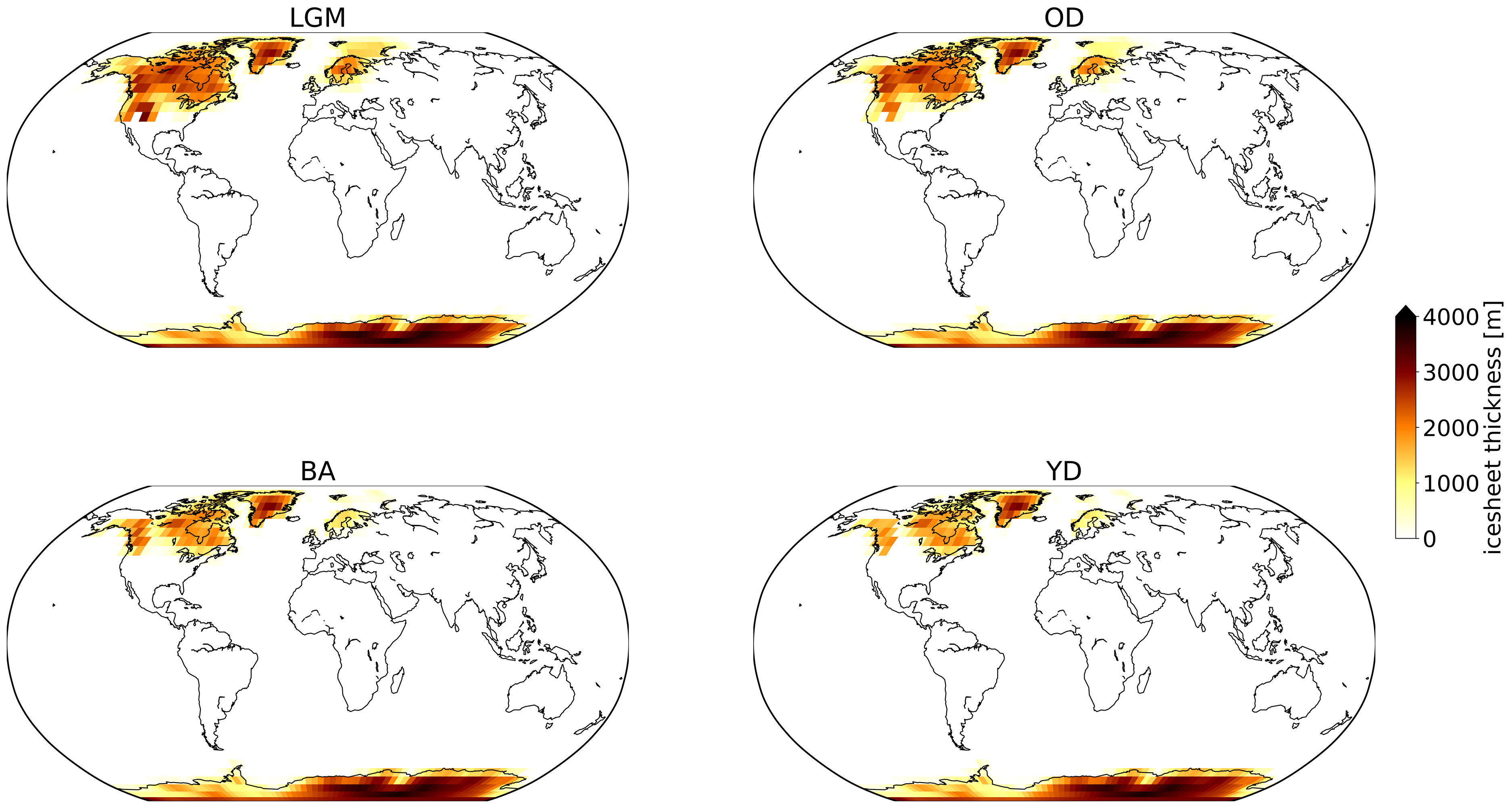

Supplement: S1 Fig — (TIF) [file pone.0300138.s001.tif]

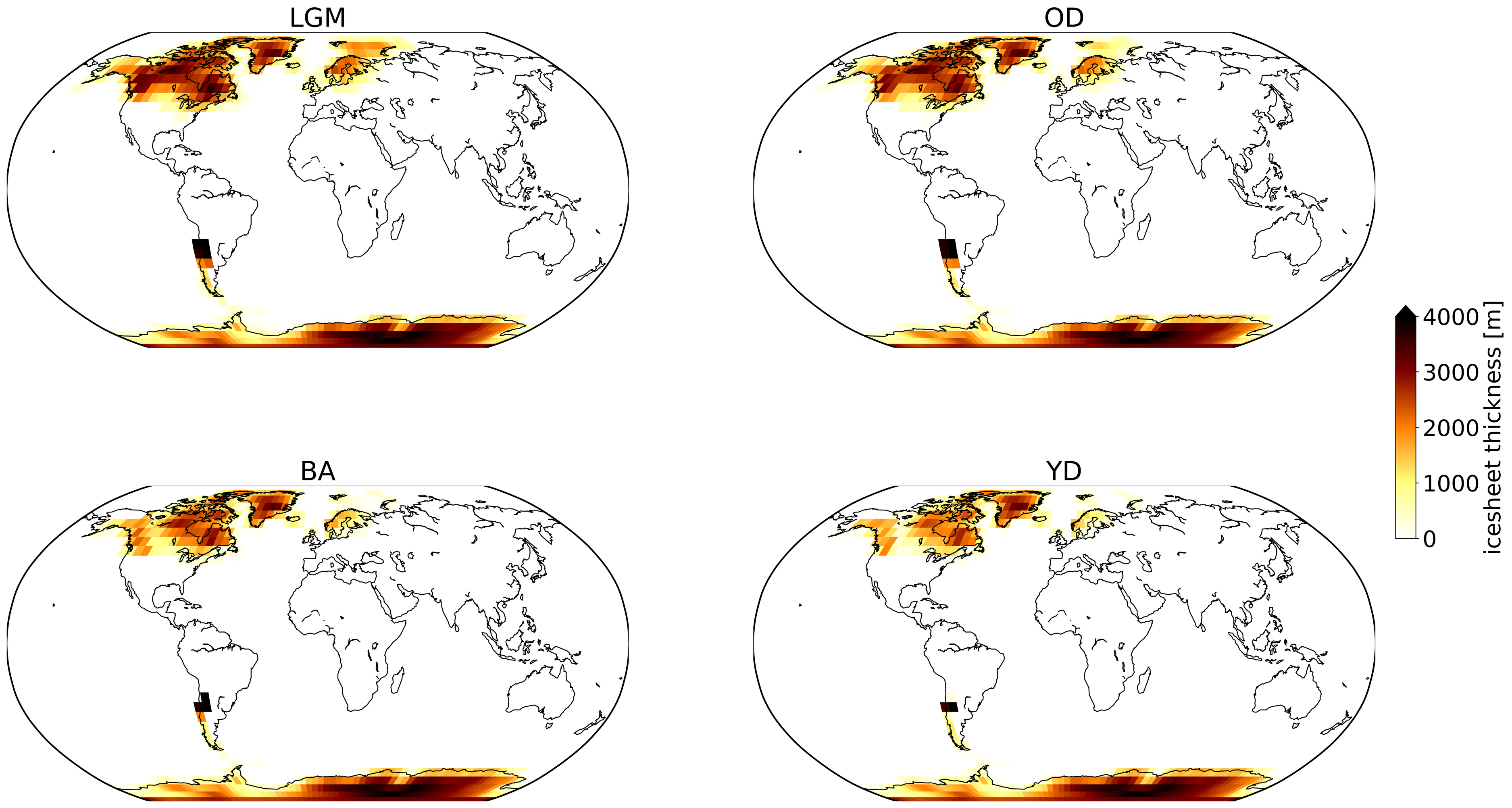

Supplement: S2 Fig — (TIF) [file pone.0300138.s002.tif]

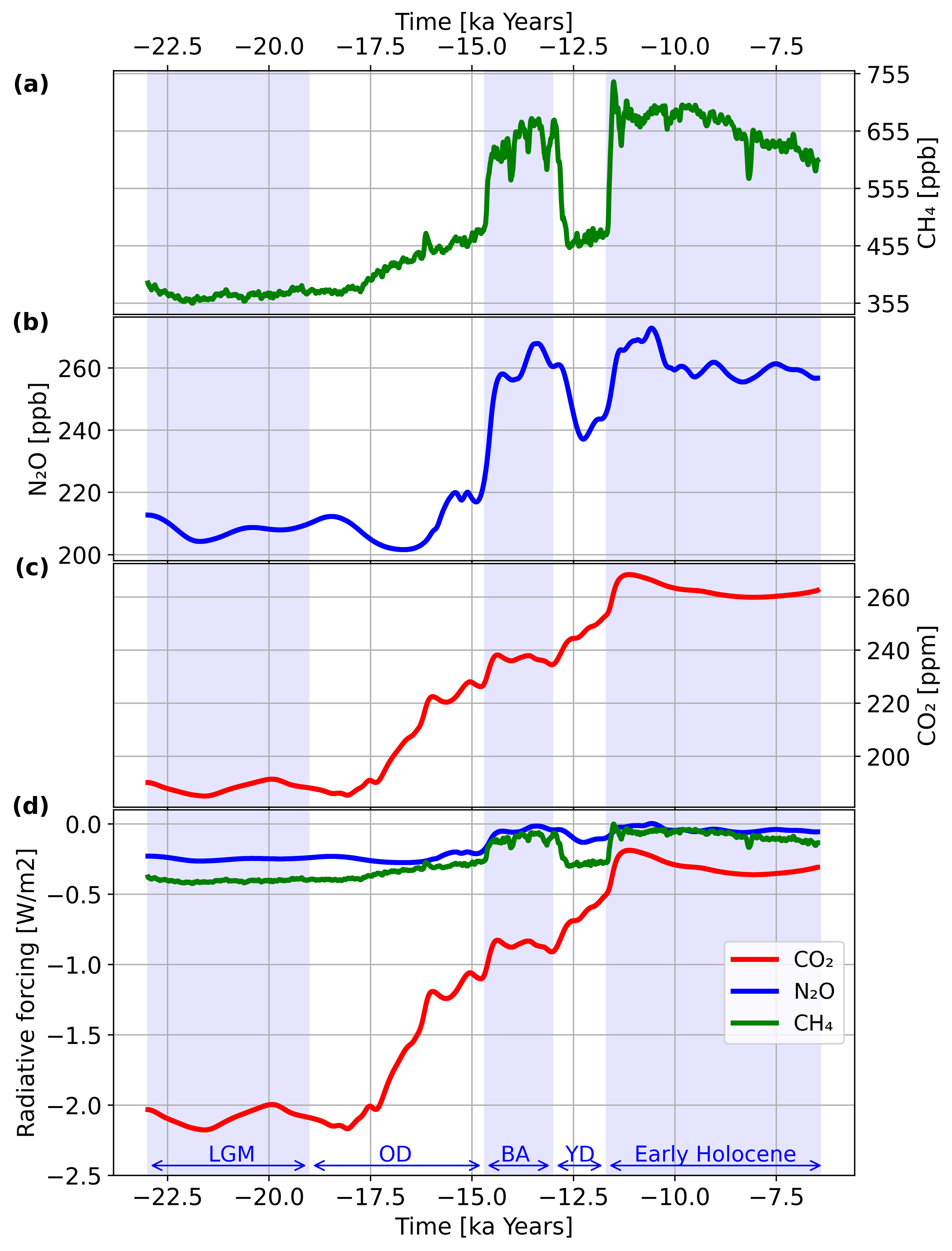

Supplement: S3 Fig — (TIF) [file pone.0300138.s003.tif]
