# Supplementary material for: Paleoclimate data assimilation with CLIMBER-X: An ensemble Kalman filter for the last deglaciation
Source: PLoS One. 2024 Apr 4;19(4):e0300138. doi: 10.1371/journal.pone.0300138 (PMC10994341; doi:10.1371/journal.pone.0300138)
Supplement: Attachment — Submitted filename: Response_to_Reviewers.pdf [file pone.0300138.s004.pdf]

Responses to Reviewers' Comments for Manuscript PONE-D-23-19461

Paleoclimate Data Assimilation with CLIMBER-X: An ensemble Kalman filter for the Last Deglaciation

Addressed Comments for Publication to

PLOS ONE

by

Ahmadreza Masoum, L. Nerger, M. Willeit, A. Ganopolski , G. Lohmann

Dear Prof. Dr. Yougui Song,

Please find enclosed the revised version of our previous submission entitled "Paleoclimate Data Assimilation with CLIMBER-X: An ensemble Kalman filter for the Last Deglaciation" with manuscript number PONE-D-23-19461. We thank you and the reviewer for the valuable comments that helped improve the quality of our manuscript. In this revision, we have carefully addressed the reviewer's comments. A summary of the main modifications and a detailed point-by-point response to the comments from the Reviewer (following the reviewers' order in the decision letter) are given below. Regarding the financial disclosure, our institute, Alfred Wegener Institute, will pay the publication costs.

Sincerely,

Ahmadreza Masoum

on behalf of all co-authors

Note: To enhance the legibility of this response letter, all the editor's and reviewers' comments are typeset in boxes.

Authors' Response to the Editor

General Comments. I hope this message finds you well. I wanted to extend my sincerest apologies for the delay in the peer review process for your manuscript. Despite our efforts, we have encountered challenges in securing potential reviewers including those you had suggested, and obtaining comments from reviewers who initially agreed to evaluate your work. By now, we only obtained one feedback, comments. Considering timeliness, we have decided to forward the comments to you. Although this is not the ideal scenario, we believe that the provided feedback can still be valuable to you for making necessary revisions to your manuscript.

Response:

Thank you for your message and sincere apologies for the challenges faced in the peer review process for my manuscript. We understand the difficulties in finding reviewers and appreciate your handling of the review process and your decision to share the obtained feedback despite the limitations. According to the reviewer's comments, we have checked our manuscript and addressed them by adding content to the Experimental Design and Discussion sections of the manuscript.

Authors' Response to Reviewer 1

General Comments. This paper, using the climate model CLIMBER-X, presents an method for online method to assimilate the temporal evolution of surface temperatures for the last deglaciation. This paper is interesting for paleoclimate reconstructions and well written. However, I have two major comments about this manuscript and it may be suitable to publish in this journal after major revisions.

Response: Thank you for your feedback and comments.

We have carefully addressed all the issues item by item as follows.

Comment 1

As we know, large amounts of paleoclimate DA methods are most based on the offline DA. That is because the observational records only provide the mean values (e.g., annual mean surface temperature) instead of instantaneous values. I think the authors should address how initial conditions change associated with annual mean surface temperature in the online DA. Additionally, it has 100 year period in the online cycle, I am not sure that the initial conditions of surface temperature is important for the surface temperature after 100 years. I think it is mainly dependent on external forcing. I suggest the authors should claim these points to increase the readability of the article.

Response:

We think this comment consists of two points: the reason for the choice of the online approach and the influence of updating initial conditions in our DA method. We believe that the main reason for the domination of the offline approach in paleoclimate DA is the high computational costs of the online DA. CLIMBER-X as an earth system model of intermediate complexity (EMIC), provides us the opportunity to apply online DA.

Moreover, the online DA approach allows us to study the performance of CLIMBER-X, including AMOC, salinity, freshwater, and many other climate parameters. Therefore, we decided to apply a more complex method, online DA, because we are able to do that, and it has the potential to get better results than offline DA. To clarify this point, We added more explanations to the manuscript, lines 164-177.

In reply to the second point, we agree that the time gap between successive observations, 100 years, may exceed the model's predictability. Thus, over 100 years we do not expect that the initial state (previous analysis) has an influence. We have added more explanations to the Discussion section, lines 375-380, and clearly mentioned that the surface temperature is mainly driven by external forcings in our experiments. However, our DA method can use high temporal resolution or artificial observations for different applications. For example, one could apply a 10-year temporal resolution just by interpolating the observation values. Then, the effect of temperature could probably persist.

Comment 2

In the manuscript, the localization radius is set to 5000 km. It seems too small. In the previous studies, they used large localization radius (such as 25000km and even infinite) to assimilate the global surface temperature. Therefore, I suggest the authors should evaluate the results according to observations and choose the best radius (shown in King et al., 2021, JC).

King et al., A Data Assimilation Approach to Last Millennium Temperature Field Reconstruction Using a Limited High-Sensitivity Proxy Network

Response:

The localization radius is always case-dependent, and there are different ways to determine an optimal radius. Depending on different factors, such as the prior states, DA approach, observation, and the targets of the articles, the previous studies have defined

their own criteria and selected different localization radii. For example, the offline DA reconstructions such as Erb et al. (2022), King et al. (2021), Osman et al. (2021), and Tierney et al. (2021) who use outputs of different coupled general circulation models for generating ensemble priors, select a relatively large radius from 12000 km to 25000 km. However, Okazaki et al. (2021) employ an EMIC for their online DA, using a 2000, 5000, and 8000 km radius.

Our main criterion is the effect of DA on the surface temperature field. During our trials, we found when we increased the radius, the North Atlantic area would be dominated by the observations located in the northwest of North America, and the effect of Greenland's observation would be lost after DA. Therefore, we decided to select a localization radius of 5000 km. Clearly, one could choose another localization radius with different observation networks. We have added further explanations in the Experimental Design, lines 183-190, and Discussion sections, lines 337-341, to address this comment and improve the readability of the paper.

References:

Tierney, J. E., Zhu, J., King, J., Malevich, S. B., Hakim, G. J., and Poulsen, C. J. (2020). Glacial cooling and climate sensitivity revisited. Nature, 584(7822):569–573.

Erb, M. P., McKay, N. P., Steiger, N., Dee, S., Hancock, C., Ivanovic, R. F., Gregoire, L. J., and Valdes, P. (2022). Reconstructing holocene temperatures in time and space using paleoclimate data assimilation. Climate of the Past, 18(12):2599–2629.

King, J. M., Anchukaitis, K. J., Tierney, J. E., Hakim, G. J., Emile-Geay, J., Zhu, F., and Wilson, R. (2021). A data assimilation approach to last millennium temperature field reconstruction using a limited high-sensitivity proxy network. Journal of Climate, 34(17):7091–7111.

Okazaki, A., Miyoshi, T., Yoshimura, K., Greybush, S. J., and Zhang, F. (2021). Revisit-

ing online and offline data assimilation comparison for paleoclimate reconstruction: an idealized osse study. Journal of Geophysical Research: Atmospheres, 126(16):e2020JD034214.

Osman, M. B., Tierney, J. E., Zhu, J., Tardif, R., Hakim, G. J., King, J., and Poulsen, C. J. (2021). Globally resolved surface temperatures since the last glacial maximum. Nature, 599(7884):239–244.
